# Sexual orientation and gender identity inequities in cervical cancer screening by race and ethnicity

**DOI:** 10.1007/s10552-023-01771-2

**Published:** 2023-08-21

**Authors:** Emmeline Lin, Patrycja Sleboda, Bobbie J. Rimel, Jarvis T. Chen, Diana V. Hernandez, Geetanjali D. Datta

**Affiliations:** 1 Cancer Research Center for Health Equity, Cedars-Sinai Medical Center, Los Angeles, CA 90069 USA; 2 Division of Gynecologic Oncology, Department of Obstetrics and Gynecology, Cedars-Sinai Medical Center, Los Angeles, CA 90048 USA; 3 Department of Social and Behavioral Sciences, Harvard T.H. Chan School Of Public Health, Boston, MA 02115 USA; 4 Department of Medicine, Cedars-Sinai Medical Center, Los Angeles, CA 90048 USA

**Keywords:** Cervical cancer, Cancer prevention, Cancer screening, Race/ethnicity, Sexual orientation, Gender identity

## Abstract

**Background:**

In the United States, inequities in preventive health behaviors such as cervical cancer screening have been documented. Sexual orientation, gender identity, and race/ethnicity all individually contribute to such disparities. However, little work has investigated their joint impact on screening behavior.

**Methods:**

Using sampling weighted data from the 2016 and 2018 Behavioral Risk Factor Surveillance System, we assessed differences in two metrics via chi-square statistics: 1) lifetime uptake, and 2) up-to-date cervical cancer screening by sexual orientation and gender identity, within and across racial/ethnic classifications.

**Results:**

Within all races, individuals who identify as members of sexual and gender minority (SGM) communities reported higher rates of never being screened (except for Black transgender men) than straight or cisgender individuals (*p* < 0.0001). [*START* Across all races, the Asian/Pacific Islander transgender population (32.4%; weighted n (*w.n.*) = 1,313) had the lowest proportion of lifetime screening, followed by the Asian/Pacific Islander gay/lesbian (53.0%, *w.n.* = 21,771), Hispanic transgender (58.7%; *w.n.* = 24,780), Asian/Pacific Islander bisexual (61.8%, *w.n.* = 54,524), and Hispanic gay/lesbian (69.6%, *w.n.* = 125,781) populations. *END*] Straight or cisgender Non-Hispanic White (*w.n.* = 40,664,476) individuals had the highest proportion of lifetime screening (97.7% and 97.5%, respectively). However, among individuals who had been screened at least once in their lifetime, identifying as SGM was not associated with a decreased proportion of up-to-date screening within or between races.

**Conclusions:**

Due to small sample sizes, especially among Asian/Pacific Islander and Hispanic populations, confidence intervals were wide. Heterogeneity in screening participation by SGM status within and across racial/ethnic groups were observed.

**Impact:**

These screening disparities reveal the need to disaggregate data to account for intersecting identities and for studies with larger sample sizes to increase estimate reliability.

**Supplementary Information:**

The online version contains supplementary material available at 10.1007/s10552-023-01771-2.

## Introduction

Cervical cancer affects thousands of people in the United States (U.S.) annually, including cis-gendered, transgender, and nonbinary individuals assigned female at birth. In 2022, 14,100 new cases of and 4,280 deaths from cervical cancer are estimated to occur in the U.S. [1]. However, cervical cancer is treatable if diagnosed early through screening. Screening is incorporated in the World Health Organization (WHO)’s plan to eliminate cervical cancer as a global public health problem, which is defined as an incidence below 4 per 100,000 person-years [2]. However, the incidence rate for new cases of cervical cancer in the U.S. is much greater than the WHO’s goal, at 7.8 per 100,000 females per year [3]. Screening guidelines are defined by the U.S. Preventive Services Task Force (USPSTF), which recommends women aged 21 to 65 to complete cervical cytology testing every 3 years, or an additional or alternative option of completing an HPV every 5 years for females aged 30 to 65 [4]. The current U.S. screening rate, 80.5%, does not yet meet the Healthy People 2030 Target of 84.3% [5] set forth by the U.S. Department of Health and Human Services. Because half of newly diagnosed cervical cancers in the U.S. occur among those who do not screen regularly, eliminating the disease will require greater screening uptake [6].

Cervical cancer screening inequities, which are inequalities deemed unfair, avoidable, or stemming from some form of injustice [7], among racial and ethnic minorities have been well-documented [8]. An analysis of data from the Behavioral Risk Factor Surveillance System (BRFSS) found that individuals from minoritized racial/ethnic groups, especially women of Asian descent, were more likely to have never been screened for cervical cancer compared to Non-Hispanic (NH) White women [9]. Additionally, only 75% of Hispanic women report receiving cervical cytology (Papanicolaou, or Pap, tests), compared to 82% in NH-White individuals [11]. Black, Hispanic, and Asian individuals are more likely to be diagnosed with advanced-stage cervical cancer than NH-White individuals [12].

Studies have also found that inequalities exist regarding sexual orientation and gender identity. The term “sexual and gender minorities” (SGM) refers to individuals who “identify as lesbian, gay, bisexual, asexual, transgender,” have “same-sex or -gender attractions or behaviors and those with a difference in sex development,” or “do not self-identify with one of these terms but…is characterized by non-binary constructs of sexual orientation, gender, and/or sex” [14]. In an analysis of the 2016 BRFSS, gay or lesbian individuals were found to have significantly decreased likelihood of adhering to cervical cancer screening recommendations compared to heterosexual individuals [15], with lesbians being over two times more likely to report never having been screened [16]. Additionally, a 2013 survey of over 5,000 women in Boston, Massachusetts found that transgender men had 37% lower odds of being up-to-date with cervical cancer screening compared to cisgender women [17]. This inequity may reflect the lack of specific screening recommendations for transgender men and nonbinary populations that still have a cervix, as the wording of current USPSTF guidelines are directed specifically towards “women” and may indirectly exclude other populations at risk.

The minority stress theory describes the unique and elevated stressors experienced by SGM groups that come from stigma and prejudice towards their SGM identity, and may contribute to the inequitable healthcare outcomes and access found among this group [18, 19]. Examples of stressors include past experiences or future expectations of prejudice and discrimination, concealment of SGM identity, and internalization of societal stigma [20]. Minority stress creates a strained relationship between SGM populations and medical providers; this relationship is characterized by distrust and fear towards, closed communication with, and avoidance of future interactions with the medical establishment [21], which may further harm this population’s health.

The intersectionality theoretical framework proposes that healthcare disparities may be exacerbated for individuals with multiple, overlapping identities, especially in societies with entrenched forms of discrimination such as racism, classism, sexism, homophobia, and transphobia [22]. These discriminatory structures and practices may act as barriers to participating in protective health behaviors such as cancer screening [23]. Therefore, individuals who are part of multiple marginalized communities are more likely to experience poorer health outcomes and more barriers to care [24–26], highlighting the necessity to consider the impact of intersecting identities when designing healthcare policies and interventions to increase screening uptake.

To our knowledge, only two studies have explored the impact of the intersection between sexual orientation and racial/ethnic identity on cervical cancer screening. A study by Stenzel et al. [23] using the 2015 and 2018 National Health Interview Survey (NHIS) reported that among racial/ethnic and sexual minority groups, Hispanic, sexual minority women had the lowest cervical cancer screening uptake compared to NH-White heterosexual women. Meanwhile, an analysis on the 2006–2010 National Survey of Family Growth (NSFG) by Agénor et al. [27] found differences in screening between heterosexual and sexual minority women only within the NH-White group, with NH-White women with female partners only reporting significantly lower odds of Pap test use compared to women with only male partners. Given the limited nature of the current literature, additional studies are necessary. Additionally, no known studies have examined differences in cervical cancer screening rates by gender identity within or across racial /ethnic categories. Therefore, the aim of the current study is to describe variation in cervical cancer screening uptake among SGM within (SGM vs. heterosexual/cisgender of the same race/ethnicity) and across (SGM of color vs. heterosexual/cisgender NH-White) racial/ethnic groups using data from the 2016 and 2018 BRFSS. We hypothesize that populations who have intersecting minority identities will have lower proportions of lifetime and up-to-date screening than NH-White, non-SGM individuals.

## Methods

### Study population

The data used in this study were obtained from the 2016 and 2018 Behavioral Risk Factor Surveillance System (BRFSS), which is an annual nation-wide telephone survey conducted by the Center for Disease Control that surveys over 400,000 U.S. residents ages ≥ 18 about their health-related risk behaviors, chronic health conditions, and use of preventive services [28]. We report data from 26 states and territories (California, Connecticut, Delaware, Georgia, Guam, Hawaii, Idaho, Illinois, Indiana, Iowa, Kentucky, Louisiana, Massachusetts, Minnesota, Mississippi, Missouri, Nevada, New York, Ohio, Pennsylvania, Rhode Island, Texas, Vermont, Virginia, Washington, and Wisconsin) that collected data on sexual orientation and gender identity (excluded unweighted *n* = 459,236).

We excluded participants who did not have a cervix (i.e., identified as cisgender male or male-to-female transgender) (unweighted *n* = 207,363), who were outside of the age range 24 to 65 (unweighted *n* = 97,653), had a hysterectomy (unweighted *n* = 26,600), answered Native-American/Alaskan-Native, multiracial, and other race due to small sample sizes (unweighted *n* = 7,617), identified as gender nonconforming since BRFSS does not explicitly survey sex assigned at birth (unweighted *n* = 175), had missing data on SGM status (unweighted *n* = 27,575), or had missing information on cervical cancer screening (unweighted *n* = 2,271). The total unweighted sample size was 98,955 individuals (Table 1).

Because the population who did not report SGM data was large, we analyzed differences in their screening behavior by racial/ethnic groups in order to confirm that the exclusion of these cases did not bias the estimates. We also examined what proportion of this nonreporting population belonged to each racial/ethnic group or screening category to determine whether disclosure of SGM status could be attributed to specific population characteristics and contribute to selection bias.

### Primary outcomes

In the U.S., cervical cancer screening recommendations are defined by the U.S. Preventive Services Task Force (USPSTF) for women. However, individuals who do not identify as female but still possess a cervix (e.g. transgender men, gender nonconforming individuals, etc.) should also be screened, as they are still at risk for developing cervical cancer [29]. Therefore, we will use the term “individuals” instead of “women” in this manuscript. When referencing previous literature, we will use the language of the cited article.

Our primary outcomes were two-fold: 1) lifetime cervical cancer screening (“Never screened” vs. "Ever screened”), and 2) up-to-date/adherent screening among those who had screened at least once in their lifetimes (“Not recently screened” vs. “Screened According to Guidelines”). We used items from the BRFSS questionnaire to ascertain screening status: “Have you ever had a Pap test?” and “Have you ever had an HPV test?” (both to which participants could respond with “Yes” or “No”), as well as “How long has it been since you had your last Pap test?” and “How long has it been since you had your last HPV test” (both to which participants could respond with “Within the past year,” “Within the past 2 years,” “Within the past 3 years,” “Within the past 5 years,” or “5 or more years ago”) [28]. Up-to-date screening was defined in accordance with the U.S. Preventive Services Task Force (USPSTF) screening guidelines [4]. Respondents aged 21 to 29 were considered adherent to recommended guidelines if they reported completing cervical cytology in the last 3 years, while respondents aged 30 to 65 were considered adherent if they reported completing a cytologic testing in the 3 years or an HPV test in the 5 years prior to the survey [4]. We limited the age range of the sample population to those over the age of 24 to account for a three-year lookback period.

We evaluated screening according to SGM and racial/ethnic minority status. Sexual orientation was categorized as straight, gay/lesbian, or bisexual, while gender identity categories included cisgender or transgender female-to-male, which we will refer to as transgender man/men. Racial/ethnic classifications included NH-White, Black, Hispanic, and Asian/Pacific Islander. Health care access hardship is defined as being unable to see a doctor within the past 12 months due to cost [30].

### Statistical analysis

Weighted and unweighted frequencies and percentages of the prevalence of each screening status within each of the demographic categories were calculated; the BRFSS provided sampling weights and stratum indicators that were used to adjust for population size [31]. Given the small sample size in some populations, e.g., Black transgender men *n* = 17, we acknowledge the limitations of upweighting, such as overrepresentation of a specific group or introduction of additional biases into the dataset [32]. Chi-square analysis was used to produce descriptive statistics and compare differences in screening adherence by SGM identity within each race/ethnic category. Missing indicators were used in Table 1 for participants who were missing responses to the covariate survey questions, which included screening predisposing factors such as marital status, educational attainment, household income relative to the federal poverty level, and health care access [34]. Stata version 17 (StataCorp, College Station, TX) was used to complete all statistical analyses. Because the goal was to describe screening behaviors according to racial/ethnic and SGM categories, we did not run any models.

## Results

### Total population characteristics

The sociodemographic characteristics of the participants included in the analytic dataset (*n =* 95,249*)* are shown in Table 1. Of the total population, 1.4% identified as gay/lesbian, 3.1% as bisexual, and 0.2% as transgender men. Over half of all respondents received at least a college education, were employed, and reported an annual family income of over 200% of the federal poverty level (FPL). Over 80% of respondents had insurance coverage and a medical exam in the last two years. Compared to heterosexual or cisgender individuals, there were a higher percentage of SGM individuals who were below FPL, had health care access hardship, and lacked a primary care physician (PCP) (non-overlapping confidence intervals). A greater proportion of transgender men also reported being unemployed and did not attend college compared to cisgender individuals. The proportion of those who reported having had a medical examination in the previous two years was similar across SGM groups (80.7–86.3%).

**Table 1:** Characteristics of individuals eligible for cervical cancer screening by sexual orientation, and gender identity. (*n* = 98,955; weighted *n* [*w.n.*] = 67,583,081)

Characteristics	% Overall Total Eligible Population (95% CI) *n* [*w.n.*]	Sexual Orientation			Gender Identity	
		% Straight (95% CI) *n* [*w.n.*]	% Gay or Lesbian (95% CI) *n* [*w.n.*]	% Bisexual (95% CI) *n* [*w.n.*]	% Cisgender Women (95% CI) *n* [*w.n.*]	% Transgender Men (95% CI) *n* [*w.n.*]
Gender Identity	98,711 [67,383,878]					
Cisgender Women	99.8 (99.7–99.8) 98,528 [67,229,741]	99.9 (99.8–99.9) 91,009 [61,231,627]	96.8 (92.7–98.6) 1,425 [948,216]	99.0 (97.1–99.6) 2,892 [2,249,405]		
Transgender Men	0.2 (0.2–0.3) 183 [154,137]	0.1 (0.1–0.2) 116 [78,489]	3.2 (1.4–7.3) 20 [34,052]	1.0 (0.4-3.0) 19 [19,942]		
Sexual Orientation	95,725 [64,760,934]					
Straight	95.5 (95.2–95.8) 91,353 [61,493,254]				95.6 (95.3–95.8) 91,009 [61,231,627]	61.4 (45.4–76.8) 116 [78,489]
Gay or Lesbian	1.4 (1.3–1.6) 1,451 [985,789]				1.4 (1.2–1.5) 20 [34,052]	22.7 (10.5–42.4) 1,425 [948,216]
Bisexual	3.1 (2.9–3.3) 2,921 [2,281,891]				3.0 (2.8–3.2) 19 [19,942]	15.8 (5.9–36.1) 2,892 [2,249,405]
Age						
24–29	14.0 (13.6–14.5) 12,398 [13,855,159]	13.3 (12.8–13.8) 10,550 [11,911,880]	23.1 (18.5–28.5) 276 [327,170]	33.9 (30.7–37.2) 1,071 [1,068,469]	14.0 (13.5–14.5) 12,323 [13,766,862]	30.4 (18.3–46.1) 44 [56,749]
30–39	26.7 (26.1–27.3) 18,482 [16,685,201]	26.2 (25.6–26.9) 16,698 [15,003,746]	26.0 (21.2–31.3) 253 [222,545]	35.8 (32.5–39.3) 815 [657,588]	26.7 (26.1–27.3) 18,398 [16,585,559]	25.0 (13.4–41.8) 28 [35,054]
40–49	23.3 (22.7–23.8) 20,366 [14,545,086]	23.4 (22.9–24.0) 18,907 [13,405,551]	22.4 (18.2–27.1) 272 [191,513]	18.3 (15.6–21.4) 503 336,143]	23.3 (22.7–23.8) 20,267 [14,463,555]	24.8 (15.7–37.0) 44 [34,745]
50–65	36.0 (35.4–36.6) 47,709 [22,497,636]	37.0 (36.4–37.6) 45,198 [21,172,077]	28.5 (24.6–32.8) 650 [244,561]	12.0 (10.2–13.9) 532 [219,691]	36.0 (35.5–36.6) 47,540 [22,413,765]	19.7 (12.3–30.0) 67 [27,589]
Race						
NH-White	61.9 (91.3–62.5) 75,400 [41,399,425]	63.3 (62.6–64.0) 70,641 [38,481,198]	66.1 (60.4–71.3) 1,135 [630,865]	69.2 (65.8–72.3) 2,226 [1,552,412]	62.0 (61.4–62.7) 75,163 [41,252,394]	52.6 (38.3–66.5) 119 [86,460]
Black	12.7 (12.2–13.1) 8,910 [8,630,929]	12.8 (12.4–13.2) 8,048 [7,942,719]	12.5 (9.5–16.2) 116 [133,196]	11.7 (9.7–14.0) 245 [261,907]	12.6 (12.2–13.1) 8,857 [8,579,246]	14.9 (6.9–29.3) 18 [21,422]
Hispanic	19.2 (18.6–19.8) 9,886 [13,193,156]	17.8 (17.2–18.5) 8,308 [11,208,353]	17.6 (13.2–23.2) 134 [180,658]	16.0 (13.5–18.9) 340 [379,270]	19.1 (18.5–19.7) 9,786 [13,085,126]	29.9 (17.9–45.4) 32 [42,208]
Asian/Pacific Islander	6.3 (5.9–6.7) 4,759 [4,359,572]	6.1 (5.7–6.5) 4,356 [3,860,983]	3.8 (2.0-7.2) 66 [41,070]	3.1 (2.1–4.5) 110 [88,302]	6.2 (5.8–6.6) 4,722 [4,312,974]	2.6 (1.0-6.4) 14 [4,047]
Education						
Elementary School or Some High School	12.8 (12.2–13.4) 5,982 [8,362,430]	11.4 (10.8–12.0) 4,823 [6,784,092]	13.1 (8.9–18.9) 66 [122,473]	9.2 (7.1–11.9) 162 [212,059]	12.7 (12.1–13.3) 5,888 [8,263,106]	25.2 (15.0-39.2) 28 [35,268]
High School	23.0 (22.5–23.6) 21,709 [15,782,640]	23.1 (22.5–23.6) 19,973 [14,329,685]	18.6 (14.9–23.0) 283 [216,141]	23.1 (20.3–26.2) 630 [549,339]	19.4 (12.1–29.8) 21,581 [15,685,517]	33.0 (23.5–44.3) 60 [37,416]
College	64.0 (63.4–64.7) 71,092 [43,327,053]	65.4 (64.7–66.1) 66,420 [40,289,505]	68.2 (62.6–73.4) 1,101 [647,057]	67.5 (64.0-70.8) 2,124 [1,517,095]	64.1 (63.5–64.8) 70,895 [43,177,833]	55.4 (41.3–68.6) 95 [81,453]
Missing	0.2 (0.1–0.2) 172 [110,958]	0.1 (0.1–0.2) 137 [89,973]	0.01 (0.002-0.1) 1 [119]	0.01 (0.02–0.9) 5 [3,398]	0.1 (0.1–0.2) 164 [103,284]	0
Marital Status						
Has a Partner	62.7 (62.0-63.3) 59,250 [40,298,772]	63.5 (62.9–64.2) 55,620 [37,294,273]	48.5 (43.1–53.8) 694 [433,377]	47.7 (44.2–51.2) 1,289 [978,059]	62.7 (62.1–63.4) 59,043 [40,135,126]	39.9 (26.8–54.6) 76 [57,049]
No Partner	36.9 (36.3–37.5) 39,282 [27,015,369]	36.1 (35.5–36.7) 35,386 [23,987,270]	51.1 (45.8–56.4) 752 [548,901]	52.0 (48.5–55.4) 1,621 [1,296,870]	60.1 (45.4–73.2) 39,065 [26,826,058]	54.6 (43.3–65.4) 107 [97,088]
Missing	0.4 (0.3–0.5) 423 [268,940]	0.3 (0.3–0.5) 347 [211,711]	0.4 (0.1–1.5) 5 [3,511]	0.4 (0.1-1.0) 11 [6,961]	0.4 (0.3–0.5) 420 [268,557]	0
Employment Status						
Employed	65.2 (64.5–65.8) 65,247 [43,382,324]	65.8 (65.2–66.5) 60,679 [39,870,547]	69.7 (64.2–74.6) 1,013 [672,746]	68.3 (64.9–71.5) 1,918 [1,525,625]	65.2 (64.6–65.8) 65,032 [43,183,441]	63.8 (50.3–75.4) 100 [94,997]
Unemployed	12.7 (12.3–13.2) 12,985 [8,462,209]	12.4 (11.9–12.8) 11,616 [7,491,735]	16.5 (13.0-20.7) 234 [159,907]	16.7 (14.3–19.4) 492 [352,973	12.7 (12.3–13.1) 12,881 [8,395,519]	11.1 (6.4–18.7) 45 [22,443]
Student/Home-maker	16.4 (15.9–17.0) 12,209 [12,148,064]	16.0 (15.5–16.6) 11,042 [10,802,654]	8.6 (5.1–14.1) 82 [106,983]	13.2 (10.7–16.0) 410 [365,348	16.4 (15.9–17.0) 12,131 [12,075,629]	23.2 (13.7–36.6) 29 [34,078]
Retired	4.9 (4.7–5.2) 7,521 [2,930,306]	5.1 (4.9–5.3) 113 [40,857]	1.2 (0.9–1.7) 83 [22,837]	4.0 (3.0-5.4) 208 [105,209]	4.9 (4.7–5.2) 7,903 [3,091,645]	0.9 (0.3–2.4) 7 [1,235]
Missing	0.7 (0.6-1.0) 495 [398,011]	0.7 (0.5–0.9) 9 [5,295]	0.5 (0.1-2.0) 18 [15,107]	0.6 (0.3–1.3) 67 [72,862]	0.7 (0.6-1.0) 581 [483,507]	1.0 (0.2–4.6) 2 [1,385]
Income as Percent of FPL						
< 100	16.8 (16.3–17.4) 12,524 [11,617,113]	15.7 (15.2–16.2) 10,808 [9,884,500]	14.9 (11.3–19.5) 172 [173,573]	20.8 (18.0-23.9) 557 [494,009]	16.7 (16.2–17.3) 12,403 [11,486,309]	40.2 (27.2–54.8) 57 [63,145]
100–200	18.2 (17.7–18.7) 18,056 [12,575,488]	18.1 (17.6–18.6) 16,465 [11,346,334]	15.0 (12.2–18.2) 269 [150,362]	22.8 (20.0-25.8) 667 [527,051]	18.2 (17.7–18.8) 17,974 [12,516,072]	15.8 (8.8–26.8) 39 [24,914]
> 200	64.9 (64.3–65.6) 68,375 [43,390,480]	66.2 (65.6–66.9) 64,080 [40,262,420]	70.1 (65.3–74.6) 1,010 [661,854]	56.4 (52.9–59.8) 1,697 [1,260,830]	65.0 (64.4–65.7) 68,151 [43,227,360]	44.0 (30.4–58.6) 87 [66,078]
Insurance Status						
Yes	87.5 (87.0–88.0) 90,490 [58,994,067]	88.6 (88.1–89.1) 84,239 [54,373,724]	89.7 (86.3–92.4) 1,324 [886,382]	84.9 (82.0-87.4) 2,584 [1,914,658]	87.5 (87.0–88.0) 90,160 [58,727,638]	81.8 (70.8–89.2) 149 [128,411]
No	12.2 (11.7–12.7) 8,212 [8,329,457]	11.1 (10.6–11.6) 6,902 [6,907,582]	10.3 (7.6–13.7) 124 [95,190]	14.8 (12.3–17.7) 326 [359,703]	12.1 (11.6–12.6) 8,118 [8,242,833]	18.1 (10.7–29.1) 33 [25,577]
Missing	0.3 (0.3–0.5) 253 [259,557]	0.3 (0.2–0.4) 212 [211,947]	0.03 (0.005-0.2) 3 [4,217]	0.3 (0.1–1.1) 11 [7,530]	0.1 (0.01–0.8) 250 [259,270]	0.3 (0.3–0.5) 1 [149]
Health Care Access Hardship						
Yes	15.6 (15.1–16.1) 12,628 [10,628,455]	14.9 (14.4–15.4) 11,110 [9,186,066]	16.2 (12.0-21.6) 197 [180,228]	28.1 (24.7–31.7) 618 [626,338]	15.5 (15.0–16.0) 12,523 [10,529,685]	37.7 (24.4–53.1) 46 [55,570]
No	84.0 (83.5–84.5) 85,937 [56,668,618]	84.7 (84.2–85.2) 79,900 [52,058,125]	83.4 (78.1–87.6) 1,249 [801,230]	71.3 (67.7–74.7) 2,289 [1,644,575]	84.0 (83.5–84.5) 85,621 [56,416,989]	62.3 (46.9–75.6) 137 [98,567]
Missing	0.4 (0.4–0.5) 390 [286,008]	0.4 (0.4–0.5) 343 [249,063]	0.6 (0.3-1.0) 5 [4,331]	0.6 (03 − 1.0) 14 [10,978]	0.4 (0.4–0.5) 384 [283,067]	0
Primary care physician						
Yes	81.4 (80.8–81.9) 83,977 [54,168,862]	82.3 (81.7–82.9) 78,239 [49,944,036]	80.1 (75.3–84.1) 1,198 [743,861]	71.9 (68.4–75.1) 2,215 [1,612,157]	81.4 (80.9–82.0) 83,652 [53,938,920]	57.2 (42.7–70.6) 133 [90,494]
No	18.1 (17.6–18.7) 14,512 [13,061,642]	17.2 (16.6–17.7) 12,704 [11,244,307]	19.2 (15.2–23.9) 245 [234,379]	27.4 (24.2–30.9) 693 [655,451]	18.0 (17.5–18.6) 14,416 [12,944,402]	39.9 (26.9–54.5) 47 [59,381]
Missing	0.5 (0.5–0.6) 466 [352,578]	0.5 (0.4–0.6) 410 [304,910]	0.7 (0.3-2.0) 8 [7,549]	0.7 (0.3–1.5) 13 [14,283]	0.5 (0.4–0.6) 460 [346,419]	3.0 (0.5–15.9) 3 [4,236]
Medical exam in the previous 2 Years						
Yes	86.2 (85.7–86.7) 28,101 [16,041,594]	86.5 (85.0-86.9) 26,629 [15,084,651]	86.3 (81.3–90.2) 369 [188,829]	81.3 (78.2–84.2) 467 [277,676]	80.7 (67.2–89.5) 28,000 [15,974,668]	86.2 (85.8–86.7) 37 [24,433]
No	12.7 (12.3–13.2) 11,731 [3,467,053]	12.4 (11.9–12.8) 11,125 [3,276,786]	16.5 (13.0-20.7) 125 [31,149]	16.7 (14.3–19.4) 179 [59,665]	12.7 (12.3–13.1) 11,695 [3,460,292]	11.1 (6.4–18.7) 15 [3,151]
Missing	1.1 (1.0-1.3) 59,123 [48,074,434]	1.0 (1.0-1.2) 53,599 [43,131,817]	1.5 (0.7–1.2) 957 [765,812]	1.4 (0.9–2.2) 2,275 [1,944,550]	1.1 (1.0-1.3) 58,833 [47,794,781]	0.9 (0.3–3.3) 131 [126,553]

Note: numbers in parentheses are CI = Confidence Interval, numbers in brackets are *w.n*.= weighted sample size

We found that individuals whose annual family income was less than the FPL, who did not receive a college education, or were unemployed (excluding students and homemakers) had lower lifetime screening rates (Suppl. Table S1). A higher proportion of individuals without a PCP or a medical examination in the past two years reported having never or not-recently screened than individuals with a PCP or recent medical exam (Suppl. Table S1).

### Screening behaviors by SGM and racial/ethnic categories separately

**Fig. 1:**
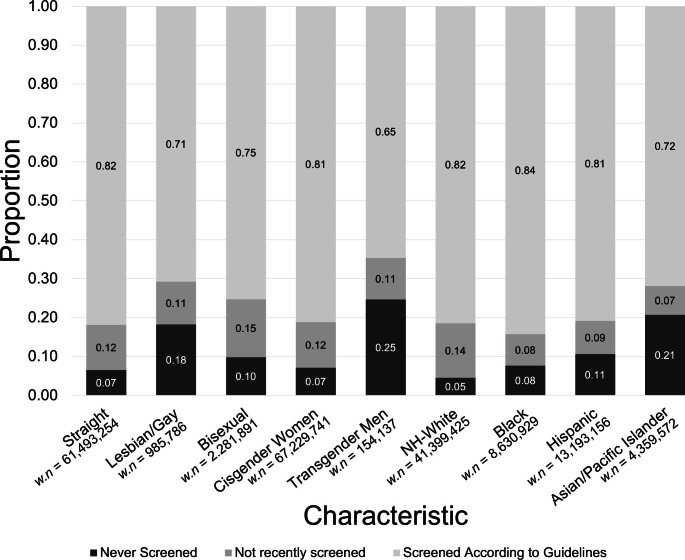
Participant characteristics by cervical cancer screening adherence (*n* = 98,955; weighted *n* (*w.n.*) = 67,583,081).

Variation in screening status was observed according to sexual and gender minority and racial/ethnic minority status, separately (Fig. 1, Suppl. Table S1). Overall, 6.5% of straight, 18.2% of gay/lesbian, 9.8% of bisexual, 7.1% of cisgender, 24.6% of transgender, 4.5% of NH-White, 7.6% of Black, 10.6% of Hispanic, and 20.7% of Asian/Pacific Islander individuals reported having never been screened for cervical cancer in their lifetime. The proportion of up-to-date screening was 81.9%, 70.8%, and 75.3% among total individuals who identified as straight, gay/lesbian, and bisexual, respectively. Moreover, 81.2% of cisgender women and 64.9% of transgender men were up-to-date with screening. Finally, 81.6% of NH-White, 84.3% of Black, 80.9% of Hispanic, and 71.9% of Asian/Pacific Islander individuals were adherent to screening guidelines.

### Screening behaviors and the Intersection of SGM status and Race/Ethnicity

Variation in screening status was observed according to sexual and gender minority and racial/ethnic minority status jointly, with proportions reported in Tables 2 and 3 and population sizes reported in **Suppl. Table S2**. Within each racial/ethnic category, those who identified as gay/lesbian had the lowest proportion of lifetime screening, while those who identified as straight had the highest proportion of lifetime screening among all sexual orientation categories (Table 2). These differences were statistically significant for all groups (p < 0.05*)*. Across all race/ethnicities, Asian/Pacific Islander gay/lesbian and bisexual individuals had the lowest rates of lifetime screening  (53.0% and 61.8%, respectively).

Among those screened in their lifetimes, those who identified as gay/lesbian or bisexual were more likely to not be screened according to guidelines. These differences were not statistically significant. The one exception to the trend described above was observed among Black gay/lesbian individuals (94.8%), where the point estimate for screening according to guidelines was higher than for their straight counterparts (91.4%). However, there was not a statistically significant difference between the groups.

Within White, Hispanic, and Asian/Pacific Islander populations, there was a higher proportion of transgender men than cisgender women who had never been screened in their lifetime (all *p* < 0.01) (Table 3); the difference was over thirty percentage points for Hispanic and Asian/Pacific Islander populations. Across racial/ethnic categories, transgender Hispanic (41.3%) and Asian/Pacific Islander (67.7%) populations had the highest proportion of respondents who had never been screened, and these proportions were greater than that of NH-White transgender men (20.5%). Respondents who identified as both Black and transgender did not have a lower lifetime screening proportion (99.8%) compared to their cisgender counterparts (92.4%). However, it should be noted that the small sample size (*n* = 17) adds uncertainty around this finding.

Among those who had been screened in their lifetime, no significant differences in screening adherence within racial/ethnic categories were observed according to gender identity (Table 3). However, sample sizes were very small (*n* as low as 12) in most groups.

**Table 2:** Screening Behavior According to Sexual Orientation, by Race (*n* = 95,725; weighted *n* (*w.n.*) = 64,760,934)

Characteristic	Screening Status						
		Never Screened		Ever Screened			
	Total *n*^a^ [*w.n.*]	% Unscreened (95% CI) *n* [*w.n.*]	*p*-value^a^	% Screened (95% CI) *n* [*w.n.*]	% Not Recently Screened ^b^ (95% CI) *n* [*w.n.*]	% Screened According to Guidelines^b^ (95% CI) *n* [*w.n.*]	*p*-value^c^
**White**	74,002 [40,664,476]	4.3 (4.0-4.6) 2,116 [1,749,841]	< 0.0001	95.7 (95.4–96.0) 71,886 [38,914,634]	14.5 (14.0–15.0) 11,423 [5,651,473]	85.5 (84.5–86.0) 60,463 [33,263,161]	0.0836
Straight	70,641 [38,481,198]	4.0 (3.7–4.3) 1,883 [1,549,295]		96.0 (95.6–96.3) 68,758 [36,931,904]	14.4 (13.9–14.9) 10,900 [5,319,989]	85.6 (85.1–86.1) 57,85 8 [31,611,915]	
Gay/Lesbian	1,135 [630,865]	13.4 (9.7–18.3) 94 [84,809]		86.6 (81.7–90.3) 1041 [546,056]	14.7 (11.7–18.4) 200 [80,306]	85.3 (81.6–88.3) 841 [465,750]	
Bisexual	2,226 [1,552,412]	7.5 (5.9–9.4) 139 [115,738]		92.5 (90.6–94.1) 2,087 [1,436,674]	17.5 (14.4–21.1) 323 [251,178]	82.5 (78.9–85.6) 1,764 [1,185,496]	
**Black**	8,409 [8,337,822]	7.4 (6.3–8.6) 476 [615,367]	0.0309	92.6 (91.4–93.7) 7,933 [7,722,456)	8.6 (7.6–9.8) 719 [665,558]	91.4 (90.2–92.4) 7,214 [7,056,898]	0.3691
Straight	8,048 [7,942,719]	7.1 (6.0-8.3) 432 [562,493]		92.9 (91.7–94.0) 7,616 [7,380,227]	8.6 (7.5–9.8) 687 [634,507]	91.4 (90.2–92.5) 6,929 [6,745,719]	
Gay/Lesbian	116 [133,196]	15.6 (8.0–28.0) 16 [20723]		84.4 (71.9–92.0) 100 [112,473]	5.2 (2.5–10.6) 10 [5,826]	94.8 (89.4–97.6) 90 [106,647]	
Bisexual	245 [261,907]	12.3 (6.3–22.5) 28 [32,151]		87.7 (77.4–93.7) 217 [229,756]	11.0 (6.0-19.3) 22 [25,225]	89.0 (80.7–94.0) 195 [204,531]	
**Hispanic**	8,782 [11,768,281]	10.7 (9.3–12.2) 682 [1,259,415]	0.0041	89.3 (87.7–90.7) 8,100 [10,508,865]	9.2 (7.4–11.3) 813 [965,260]	90.8 (88.7–92.6) 7,287 [9,543,605]	0.0337
Straight	8,308 [11,208,353]	10.4 (9.0-11.9) 616 [1,162,333]		89.6 (88.0–91.0) 7,692 [10,046,020]	8.9 (7.6–10.4) 763 [896,578]	91.1 (89.6–92.4) 6,929 (9,149,442)	
Gay/Lesbian	134 [180,658]	30.4 (13.8–54.3) 26 (54,877)		69.6 (45.7–86.2) 108 [125,781]	15.5 (8.1–27.7) 17 [19,479]	84.5 (72.3–92.0) 91 [106,302]	
Bisexual	340 [279,270]	11.1 (7.0-17.2) 40 [42,206]		88.8 (82.8–93.0) 300 [337,064]	14.6 (9.3–22.2) 33 [49,203]	85.4 (77.8–90.7) 267 [187,861]	
**Asian/Pacific Islander**	4,532 [3,990,355]	18.8 (16.5–21.4) 719 [750,512]	0.0007	81.1 (78.6–83.5) 3813 [3,239,843]	9.2 (7.9–10.6) 414 [298,179]	90.8 (89.4–92.1) 3,399 [2,941,664]	0.0438
Straight	4,356 [3,860,983]	18.1 (15.7–20.7) 672 [697,436]		81.9 (79.3–84.3) 3,684 [3,163,547]	8.9 (7.2–11.1) 398 [282,197]	91.1 (88.9–92.8) 3,286 [2,881,350]	
Gay/Lesbian	66 [41,070]	47.0 (21.8–73.8) 17 [19,299]		53.0 (26.2–78.2) 49 [21,771]	11.2 (3.2–32.6) 6 [2,446]	88.8 (67.4–96.8) 43 [19,325]	
Bisexual	110 [88,302]	38.3 (24.1–54.7) 30 [33,778]		61.8 (45.3–75.9) 80 [54,524]	24.8 (9.2–51.7) 10 [13,536]	75.2 (48.3–90.8) 70 [40,989]	

Note: numbers in parentheses are CI = Confidence Interval, numbers in brackets are *w.n*.= weighted sample size

**Table 3:** Screening Behavior According to Gender Identity, by Race (*n* = 98,711; weighted *n* (*w.n.*) = 67,383,878)

Characteristic	Screening Status						
		Never Screened		Ever Screened			
	Total *n*^a^ [*w.n.*]	% Unscreened (95% CI) *n* [*w.n.*]	*p*-value^a^	% Screened (95% CI) *n* [*w.n.*]	% Not Recently Screened^b^ (95% CI) *n* [*w.n.*]	% Screened According to Guidelines^b^ (95% CI) *n* [*w.n.*]	*p*-value^c^
**White**	75,282 [41,338,855)	4.4 (4.1–4.8) 2,234 [1,840,664)	< 0.0001	95.6 (95.2–95.9) 73,048 [39,498,191]	14.6 (14.1–15.1) 11,681 [5,777,482]	85.4 (84.9–85.9) 61,367 [33,720,709]	0.3247
Cisgender Women	751,163 [41,252,394)	4.4 (4.1–4.8) 2,218 [1,822,927)		95.6 (95.3–95.9) 72,945 [39,429,467]	14.6 (14.1–15.1) 11661 [5763697]	85.2 (84.9–85.9) 61,284 [33,665,770]	
Transgender Men	119 [86,460)	20.5 (10.0-37.4) 16 [17,737)		79.5 (62.6–90.0) 103 [68,723]	20.1 (10.5–35.0) 20 [13,784]	79.9 (65.0-85.9) 83 [54,939]	
**Black**	8875 [8,600,668]	7.6 (6.6–8.8) 518 [654,399)	< 0.0001	92.4 (91.2–93.4) 8,357 [7,946,269]	8.7 (7.7–9.9) 765 [692,905]	91.3 (90.1–92.3) 7,592 [7,523,364]	0.2986
Cisgender Women	8,857 [8,579,246]	7.6 (6.6–8.8) 517 [654,348)		92.4 (91.2–93.4) 8,340 [7,924,898]	8.7 (7.7–9.9) 764 [692,228]	91.3 (90.1–92.3) 7,576 [7,232,670]	
Transgender Men	18 [21,422]	0.23 (.03-1.9) 1 [50.73)		99.8 (98.1–100.0) 17 [21,371]	3.2 (0.4–21.0) 1 [676.7]	96.8 (78.6–99.6) 16 [20,694]	
**Hispanic**	9,818 [13,127,335]	10.6 (9.3–12.0) 774 (1,387,335)	0.0009	89.4 (88.0-90.7) 9,044 [11,739,980]	9.4 (8.2–10.7) 946 [1,104,810]	90.6 (89.3–91.8) 8,098 [10,635,170)	0.8569
Cisgender Women	9,786 [13,085,126]	10.5 (9.2–11.9) 766 [1,369,926]		89.5 (88.1–90.8) 9,020 [11,715,200]	9.4 (8.2–10.7) 943 [1,102,775]	90.6 (89.3–91.8) 8,077 [10,612,426]	
Transgender Men	32 [42,208]	41.3 (17.6–69.9) 8 [17,428]		58.7 (30.1-82.5) 24 [24,780]	8.2 (1.7–31.0) 3 [2,036]	91.8 (69.0-98.3) 21 [22,744]	
**Asian/Pacific Islander**	4736 [4,317,021]	20.8 (18.4–23.4) 799 [896,517]	0.0010	79.2 (76.6–81.7) 3,937 [3,420,504]	9.2 (7.4–11.2) 429 [313,075]	90.8 (88.8–92.6) 3,508 [3,107,429]	0.5206
Cisgender Women	4,722 [4,312,974]	20.7 (18.3–23.4) 791 [893,783]		79.3 (76.6–81.7) 3,931 [3,419,191)	9.2 (7.4–11.2) 429 [313,075]	90.8 (88.8–92.6) 3,502 [3,106,116]	
Transgender Men	14 [4,047]	67.7 (32.6–90.0) 8 [2,734]		32.4 (10.0-67.4) 6 [1,313)	0	1 (100.0) 6 [1,313]	

Note: numbers in parentheses are CI = Confidence Interval, numbers in brackets are *w.n*.= weighted sample size

^a^Chi-square among all participants; individuals who have ever been screened in their lifetime compared with women who have never been screened in their lifetime

^b^These proportions are calculated as the percent out of the total, *% Screened*

^c^Chi-square among individuals who have ever been screened in their lifetime; individuals who adhered to screening guidelines (recently screened) compared with individuals who have screened but not according to guidelines (not recently screened)

### Screening behavior for population that did not report sexual orientation or gender identity

We analyzed the screening behavior within racial/ethnic groups among the population without SGM responses, to identify any potential systematic differences between the two groups (**Table S2**). While screening proportions within each race and overall racial demographics were similar between the SGM and non-SGM-reporting populations (**Table S2**) (differences ± 2%/overlapping confidence interval), we cannot make definitive conclusions about selection bias.

## Discussion

We analyzed BRFSS data using sampling weights to assess variation in cervical cancer screening according to sexual and gender minority status and racial/ethnic categories in the U.S. Given that most cases of invasive cervical cancers in the U.S. result from either a lack of screening, underscreening, or failure to follow-up on abnormal screen results [35], these disparities have important implications for survival. We found that within the total population, sexual, gender, and racial/ethnic minorities independently had a lower rate of lifetime screening and adherence to screening guidelines compared to those who identified as heterosexual, cisgender, and NH-White individuals, respectively. When race/ethnicity and SGM status were assessed jointly, within each racial/ethnic category, SGM individuals had a significantly lower proportion of lifetime screening compared to non-SGM individuals of the same race (all *p* < 0.05). Across races, we found that NH-White, SGM individuals had higher proportions of lifetime screening than SGM individuals who also identified within a racial/ethnic minority except for Black, transgender men. We also found that among all SGM individuals, Hispanic or Asian/Pacific Islander populations had the lowest proportion of lifetime screening participation.

When assessing adherence to screening guidelines, we observed that within Black and Hispanic populations, a smaller proportion of gay/lesbian individuals were up-to-date with screening than those who were straight, while for the Hispanic population, a lower proportion of transgender men were up-to date with screening than cisgender women. Overall, differences in screening behavior within races were not statistically significant, and these findings suggest that programs aimed at reducing inequalities in cervical cancer screening should focus on increasing lifetime screening rates in all populations.

Among the Black and Asian/Pacific Islander populations, transgender men had a higher proportion of up-to-date screening than cisgender individuals, a discrepancy most likely due to small sample size, which is indicative of these populations’ participation in research or in openness about SGM identities. While Asian and Black communities are not monoliths, research suggests that Asian Americans are less willing or likely to participate in health research compared to other racial/ethnic groups, often due to language barriers or insufficient recruitment efforts by researchers [36]. Moreover, the base rate of individuals who are both Asian and SGM status is very low [37] potentially because members of some Asian communities delay or suppress acknowledgment of their SGM identities [38] due to social stigma and cultural norms, such as filial piety and family obligations [39]. Meanwhile, some Black populations have been found to have low rates of research participation due to factors such as distrust from historical research abuse or institutional racism/structural oppression, and financial barriers [40]. Black individuals are also less likely than NH-White individuals to openly disclose their sexuality due to the social stigma in the African American community towards SGM identities [41], further decreasing their representation in research.

These results should also be interpreted in the context of the two prior papers [23] that have investigated the intersectionality of SGM with racial/ethnic minorities. The heterogeneity in screening rates within racial/ethnic groups, especially as documented across studies of Black and Hispanic individuals, highlights the need to assess screening while considering intersecting identities. This current study finds that the lowest proportion of lifetime screening was reported among individuals identifying as both Asian/Pacific Islander and SGM, who were not included in prior research due to the small sample size of the Asian population [23]. Similar to the previous study [23], however, we observed that out of all intersecting populations, Hispanic, sexual minority women were among those with the lowest rates of lifetime screening. We also observed a lower proportion of lifetime screening among individuals who were both Black and gay/lesbian or bisexual than those who were both NH-White and straight; such differences were not found in prior work [23].

The current results have some similarities and some differences with a recent analysis of the 2016 BRFSS focused on SGM populations and screening behaviors conducted by Charkhchi et al. [15], which found that gay/lesbian individuals were less likely to adhere to cervical cancer screening recommendations compared to heterosexual individuals. However, Charkhchi et al. [15] combined both unscreened individuals and non-up-to-date-individuals into a singular non-adherent population to compare with the adherent population. In this paper we added a second outcome, lifetime screening, which revealed differences in types of screening behavior among different populations. We found that lifetime screening was lower among SGM individuals compared to straight or cisgender individuals, respectively, while there were no significant differences in screening non-adherence between the two comparisons, that is straight vs. sexual minority and cisgender vs. transgender men (for details see Suppl. Table S1). Additionally, we stratified the data by a second level using race/ethnicity, which revealed more specific differences in lifetime screening. The results of our study show that among all gay/lesbian or bisexual individuals, as well as for transgender men, rates for lifetime screening varied across racial groups, with the lowest rates being reported among Asians/Pacific Islanders, followed by Hispanics. However, no statistically significant differences were observed among those who had been screened in their lifetime, but not according to guidelines. Together, these differences in study design highlights the necessity to disaggregate data when analyzing vulnerable populations, which is especially important when considering how women who have never been screened are at higher risk of being diagnosed with invasive cancer than women who have been screened previously [42–44].

It is important to consider the reasons behind non-participation or non-adherence to cervical cancer screening recommendations when addressing this issue. One reason is medical mistrust [10, 12, 45–48], which develops in response to both the systemic and individual-level oppression and discrimination (e.g., racism, sexism, homophobia, transphobia, etc.) perceived by patients in a healthcare setting, and leads to greater hesitancy towards and reduced engagement with medical establishment [49]. This creates barriers for access and delay in utilization of health care services [51], which contributes to deterioration of health [52]. Past studies have shown a negative association between medical mistrust and cancer screening adherence, especially among Black and Hispanic individuals [53], due to a history of abuse by the medical system, fear of racist treatment, and concerns that doctors are more concerned with earning money than taking care of their patients [55].

Medical mistrust driven by non-affirming care and traumatic patient-provider interaction also affects healthcare access and cancer screening uptake among SGM patients [48, 56]. SGM patients report difficulty finding providers who will treat them with dignity and respect, as well as greater rates of discrimination or invalidation of their identity (e.g., not using preferred pronouns) compared to non-SGM patients [57–59]. As a result, SGM individuals more likely to delay needed care, including cervical cancer screening, due to past negative experiences in comparison to non-SGM women [22, 48, 60]. For SGM individuals who do routinely visit a healthcare provider, hesitation to disclose their identity due to fear of discrimination during the visit serves as an additional barrier to care [61]. This may be indirectly harmful to these patients’ health [62] when physicians are unable to provide the most relevant or appropriate medical recommendations, such as screening for cancer [63].

Other reasons for low uptake of cervical cancer screening among racial/ethnic minority groups include language barriers [45], not understanding that screening was necessary [46], fear of discomfort and pain [46], being too busy [65], having undocumented immigration status [66], and being unable to afford high-quality or continuous medical care due to lower socioeconomic status (SES) [67–69]. Barriers related to SES also impact screening behavior among SGM groups; compared to both the general U.S. and non-SGM populations, SGM individuals are disproportionately poor^70–72^ and are more likely to lack health insurance [26, 73–75] or have plans with inadequate coverage [76]. This translates to greater healthcare access hardship among SGM populations; lesbian and bisexual females [26, 73, 77–79] and transgender men [26] are more likely to lack a primary/routine source of healthcare and forgo/delay medical care due to cost compared to heterosexual and cisgender individuals, respectively. Similarly, racial/ethnic minorities, who experience poverty at higher rates than NH-White populations [67], are more likely to lack insurance [81] and continuity of care [67]. These previous findings and the data presented in Table 1 illustrate how marginalized groups formed by societal systems of oppression and privilege (e.g., classism, racism, homophobia/transphobia) can experience greater barriers to care compared to nonmarginalized groups. The intersectionality framework suggests that individuals who occupy multiple marginalized groups (i.e. an SGM individual who is also a member of a minoritized racial/ethnic group) experience multilevel and multifaceted barriers to screening and preventive behaviors [23–26], including for example lower SES, less access to high quality health insurance, lack of transportation, and other individual, provider, or system-level factors. Therefore, interventions to increase screening uptake must consider multiple aspects of an individuals’ identity. This can begin with clinicians carefully listening to members of vulnerable populations as they describe their needs and experiences and may be aided by the development and routine utilization of a reliable, standardized measurement tool—for example, survey or scales inquiring about identities and barriers—during clinic visits, allowing physicians to gain a clearer understanding of the specific challenges faced by each patient and population group. One such scale for cervical screening and Pap tests has been modified from the Champion’s Health Belief Model (CHBM) scale [69], which was originally validated for breast cancer screening and measures susceptibility, seriousness, benefits and barriers (inconvenient, expensive, unpleasant, painful or upsetting) [82]. Ultimately, however, efforts should also include engagement with community advocates, public health practitioners, and policy makers who can identify and address structural barriers to health equity at the societal level.

Confusion over the recommendation guidelines themselves is another potential reason for not engaging in cervical cancer screening. Screening guidelines are generally gendered in their language. However, for those who are transgender or gender nonconforming, this can lead to misunderstandings about in which screening to engage. To minimize ambiguity, where possible, organ-specific guidelines as opposed to sex- or gender-specific guidelines should be detailed [83]. Current cervical cancer screening guidelines from the American Cancer Society use the term “individuals,” while the American Society of Colposcopy and Cervical Pathology guidelines for management of cervical cancer screening abnormalities use the term “patients,” which are both gender-nonspecific. However, the USPSTF screening guidelines still utilize “women,” which could cause confusion among some members of the transgender or gender nonconforming communities.

Novel screening modalities, such as HPV self-sampling, should be explored as ways to increase screening uptake. Such tests could be completed in clinical settings or at-home [35]. Because the samples are collected by the individuals themselves, many of the known barriers to screening can be surmounted [84–86]. Several jurisdictions [87–89] have already implemented programs using self-sampled HPV tests to reach those who are underscreened, and lessons learned from those locations may be useful in informing interventions aimed at increasing screening uptake among the underscreened in the U.S. as well.

This study is subject to some limitations. Despite combining datasets from two years, sample sizes for certain intersectional groups were small, such as for the Black transgender population. Small sample sizes reduce the power of a study and increase the likelihood of reporting a false negative; this may also explain why we report that significant differences in screening adherence were not found [90]. These small sample sizes resulted in wide confidence intervals for all reported estimates, especially in the Black and Asian/Pacific Islander populations, which decrease the reliability and precision of our estimates and therefore should not be used to make strong conclusions but rather to design larger confirmatory studies [91]. This small sample size also inhibits evaluation of the intersection of both sexual orientation and gender identity on screening uptake by race, as this sub-stratification renders this population size insufficient for analysis (e.g. n_(Black, lesbian, transgender men)_ = 1). According to the intersectionality framework, the more systems of oppression—in this case, the triad of racism, homophobia, and transphobia—an individual experiences, the greater the negative effect on their health, emphasizing the need to address health disparities among this particular group [24–26]. Additionally, the small sample size results from excluding participants with missing data, who are more likely to be part of marginalized communities such as SGM groups who may be hesitant to disclose their status due to fear of discrimination [92]. As a result, our small sample size may be exclusive of the very population we are interested in. To make research findings more conclusive, future work should include datasets with larger samples of, and research efforts should focus on increasing study participation among, individuals with intersecting identities. This also applies to Native-Indian/Alaskan-Native and multiracial populations, which were excluded from this study but are often combined into one category labeled “Other Race” in other studies [15, 23, 27]. By not examining the health behaviors for these smaller groups individually, crucial information needed to tailor specific interventions remain absent from literature, contributing further to the health disparities experienced by specific racial/ethnic groups [93] or intersecting SGM populations [95].

A third limitation focuses on the validity of the self-reported screening outcomes. Because women, especially those who are lower-income or non-NH-White, tend to over-report cervical cancer screening uptake within a given time frame [97, 99], the proportion of individuals who were not up-to-date with screening may have been underreported [10]. Additionally, the absence of a statistically significant inequality in our data on differences in screening adherence could be due to the exclusion of an unknown variable affecting the participant selection process [10].

Fourth, though the BRFSS aims to be representative of the general population, it is unlikely to be representative of vulnerable sub-populations [100–103]. Not only did we only include data from states that asked questions about gender and sexual identity (approximately half of the U.S.), the proportion of our population that identified as each SGM group was also lower than that reported by the U.S. Census Bureau, which found that for adults assigned female at birth, 2.2% identified as gay/lesbian, 5.9% identified as bisexual, and 0.6% identified as transgender [104]. Furthermore, the broad categorization of socio-demographic categories, such as race/ethnicity, creates potential to overlook the heterogeneity within the sub-populations. For example, different nationalities within the Hispanic [105] or Asian/Pacific Islander [106] populations differ in poverty rates and median family income; as a social determinant of health, socioeconomic status affects health behaviors and outcomes, and is associated with lower rates of cervical cancer screening [35]. This also applies to SGM groups—where this data is recorded, such as the BRFSS, many sub-categories of sexual orientation are not accounted for (e.g. pansexual, asexual, etc.) [28], creating a gap in data on important populations.

## Conclusion

Our findings demonstrate that cervical cancer screening for eligible individuals differs according to SGM status within and across race/ethnicity, especially for those who are members of intersecting minority subpopulations. Specifically, gay/lesbian or transgender Hispanic and Asian/Pacific Islander individuals had the lowest rates of lifetime screening participation, while all SGM individuals from all racial minority groups had lower rates of lifetime participation than straight, NH-White individuals. Within all races, SGM individuals had lower rates of lifetime screening participation (except for Black transgender and Hispanic bisexual) and guideline screening adherence (except Asian/Pacific Islander transgender) than straight/cisgender individuals. Future work should continue disaggregating data to account for intersecting identities when examining the disparities in cervical cancer screening, measured as either lifetime participation or up-to-date adherence, in order to promote the establishment of culturally-competent healthcare interventions and better target groups in need.

### Electronic Supplementary Material

Below is the link to the electronic supplementary material.

Supplementary Material 1

## Data Availability

The original 2016 and 2018 BRFSS datasets utilized during the current study are available online via the Centers for Disease Control website, 2016 and 2018.
