# Supplementary material for: Sexual orientation and gender identity inequities in cervical cancer screening by race and ethnicity
Source: Cancer Causes Control. 2023 Aug 21;35(1):133–51. doi: 10.1007/s10552-023-01771-2 (PMC10764448; doi:10.1007/s10552-023-01771-2)
Supplement: Supplementary file 1 — Supplementary Material 1 [file 10552_2023_1771_MOESM1_ESM.pdf]

Table S1. Prevalence of lifetime never screening and non-recent screening by demographic and socioeconomic characteristics among eligible individuals from 2016 and 2018 BRFSS.

Variable	Population Proportion	*n* (Weighted *n*)	% Never Screened (95% CI)	% Non-recently Screened (95% CI)
Total			7.1 (6.7-7.5)	11.7 (11.3-12.1)
Gender Identity				
Cisgender	99.8 (99.7-99.8)	98,528 (67,229,741)	7.1 (6.7-7.4)	11.7 (11.3-12.1)
Transgender Man	0.2 (0.2-0.3)	183 (154,137)	24.6 (14.6-38.4)	10.7 (6.0-18.2)
Sexual Orientation				
Straight	95.5 (95.2-95.8)	91,353 (61,493,254)	6.5 (6.1-6.8)	11.6 (11.2-12.0)
Gay or Lesbian	1.4 (1.3-1.6)	1,451 (985,789)	18.2 (13.3-24.5)	11.0 (8.8-13.5)
Bisexual	3.1 (2.9-3.3)	2,921 (2,281,891)	9.8 (8.1-11.8)	14.9 (12.5-17.5)
Age				
24-29	14.0 (13.6-14.5)	12,398 (13,855,159)	20.6 (19.3-22.0)	8.0 (7.0-9.1)
30-39	26.7 (26.1-27.3)	18,482 (16,685,201)	4.6 (3.9-5.4)	7.7 (7.0-8.3)
40-49	23.3 (22.7-23.8)	20,366 (14,545,086)	3.7 (3.1-4.3)	11.2 (10.4-12.1)
50-65	36.0 (35.4-36.6)	47,709 (22,497,636)	2.9 (2.5-3.3)	17.4 (16.7-18.0)
Race				
White	61.9 (91.3-62.5)	75,400 (41,399,425)	4.5 (4.1-4.7)	14.0 (13.5-14.5)
Black	12.7 (12.2-13.1)	8,910 (8,630,929)	7.6 (6.6-8.8)	8.1 (7.1-9.1)
Hispanic	19.2 (18.6-19.8)	9,886 (13,193,156)	8.5 (7.4-9.6)	10.6 (9.3-12.0)
Asian	6.3 (5.9-6.7)	4,759 (4,359,572)	20.7 (18.3-23.4)	7.4 (6.0-9.0)
Education				
Elementary School or Some High School	12.8 (12.2-13.4)	5,982 (8,362,430)	10.5 (8.8-12.4)	14.0 (12.5-15.6)
High School	23.0	21,709	8.8	14.9

	(22.5-23.6)	(15,782,640)	(7.9-9.7)	(14.0-15.7)
College	64.0	71,092	5.8	10.2
	(63.4-64.7)	(43,327,053)	(5.4-6.2)	(9.7-10.6)
Missing	0.2	172	15.2	13.0
	(0.1-0.2)	(110,958)	(7.5-28.6)	(8.0-20.4)
Marital Status				
Has a Partner	62.7	59,250	3.7	11.2
	(62.0-63.3)	(40,298,772)	(3.3-4.0)	(10.7-11.7)
No Partner	36.9	39,282	12.2	12.4
	(36.3-37.5)	(27,015,369)	(11.4-13.1)	(11.8-13.0)
Missing	0.4	423	6.9	27.3
	(0.3-0.5)	(268,940)	(3.7-12.3)	(14.1-46.2)
Employment Status				
Employed	65.2	65,247	5.5	10.6
	(64.5-65.8)	(43,382,324)	(5.1-6.0)	(10.1-11.1)
Unemployed	12.7	12,985	9.0	17.2
	(12.3-13.2)	(8,462,209)	(7.9-10.3)	(16.0-18.4)
Student/Homemaker	16.4	12,209	12.4	9.7
	(15.9-17.0)	(12,148,964)	(11.2-13.8)	(8.9-10.5)
Retired	4.9	7,925	2.3	18.9
	(4.7-5.2)	(3,099,210)	(1.7-3.1)	(17.2-20.6)
Missing	0.7	589	13.0	22.7
	(0.6-1.0)	(491,275)	(7.9-20.5)	(10.1-43.5)
Income as Percent of FPL				
<100	16.8	12,524	10.5	12.9
	(16.3-17.4)	(11,617,113)	(9.2-11.8)	(11.9-14.0)
100-200	18.2	18,056	8.4	13.9
	(17.7-18.7)	(12,575,488)	(7.5-9.4)	(13.0-14.9)
>200	64.9	68,375	5.8	10.8
	(64.3-65.6)	(43,390,480)	(5.4-6.2)	(10.3-11.3)
Insurance Status				
Yes	87.5	90,490	6.0	10.7
	(87.0-88.0)	(58,994,067)	(5.7-6.4)	(10.4-11.1)
No	12.2	8,212	14.0	18.7
	(11.7-12.7)	(8,329,457)	(12.2-15.9)	(17.2-20.3)
Missing	0.3	253	32.6	13.8
	(0.3-0.5)	(259,557)	(20.8-47.1)	(8.3-22.0)
Health Care Access Hardship due to Cost				

Yes	15.6 (15.1-16.1)	12,628 (10,628,455)	8.6 (7.5-9.8)	18.4 (17.1-19.8)
No	84.0 (83.5-84.5)	85937 (56,668,618)	6.8 (6.4-7.2)	10.5 (10.1-10.9)
Missing	0.4 (0.4-0.5)	390 (286,008)	14.1 (8.3-23.0)	12.1 (8.4-17.0)
Personal Doctor				
Yes	81.4 (80.8-81.9)	83,977 (54,168,862)	5.2 (4.8-5.5)	10.2 (9.8-10.6)
No	18.1 (17.6-18.7)	14512 (13,061,642)	15.0 (13.7-16.5)	18.0 (16.9-19.2)
Missing	0.5 (0.5-0.6)	466 (352,578)	12.3 (7.7-18.9)	15.7 (11.1-21.6)
Checkup in the Last 2 Years				
Yes	86.2 (85.7-86.7)	86,714 (58,058,263)	6.0 (5.6-6.4)	8.6 (8.2-9.0)
No	12.7 (12.3-13.2)	11,215 (8,768,080)	13.6 (12.1-15.1)	31.7 (30.2-33.4)
Missing	1.1 (1.0-1.3)	1,026 (756,739)	17.2 (12.7-22.9)	18.9 (15.3-23.0)

Note: CI=Confidence Interval

Table S2. Screening behavior among individuals who did (SGM Data) and did not (No SGM Data) report SGM status.

Race	% Total *n*[a] (95% CI) *n* [*w.n.*]		Screening Status: Never Screened: % Unscreened (95% CI) *n* [*w.n.*]		Ever Screened: % Screened (95% CI) *n* [*w.n.*]		% Not Recently Screened (95% CI) *n* [*w.n.*]		% Screened According to Guidelines (95% CI) *n* [*w.n.*]	
	**SGM Data**	**No SGM Data**	**SGM Data**	**No SGM Data**	**SGM Data**	**No SGM Data**	**SGM Data**	**No SGM Data**	**SGM Data**	**No SGM Data**
**Total**	98,955 [67,583,081]	21,370 [26,786,357]	7.1 (6.7-7.5) 4,350 [4,800,748]	8.0 (7.1-8.9) 1,233 [2,136,946]	92.9 (92.5-93.3) 9,460 [62,782,333]	92.0 (91.1-92.8) 20,137 [24,649,411]	12.6 (12.2-13.1) 13,864 [54,856,007]	12.5 (11.7-13.4) 2,890 [3,087,737]	87.4 (86.9-87.8) 80,741 [54,856,007]	87.5 (86.6-88.3) 17,247 [21,561,675]
**White**	61.9 (91.3-62.5)	55.0 (0.54-0.56)	4.3 (4.0-4.6)	5.4 (0.05-0.06)	95.7 (95.4-96.0)	94.6 (0.94-0.95)	14.5 (14.0-15.0)	14.1 (0.13-0.15)	85.5 (84.5-86.0)	85.9 (0.88-0.93)

	74,002	15,263	2,242	645	73,158	14,618	11,705	2,286	61453	12,332
	[40,664,476]	[14,732,441]	[1,843,789]	[801,406]	[39,555,635]	[13,931,035]	[5,787,600]	[1,961,617]	[33,768,03]	[11,969,419]
**Black**	12.7	11.7	7.4	7.5	92.6	92.5	8.6	9.1	91.4	90.9
	(12.2-13.1)	(0.11-0.13)	(6.3-8.6)	(0.05-0.11)	(91.4-93.7)	(0.89-0.95)	(7.6-9.8)	(0.07-0.12)	(90.2-92.4)	(0.88-0.93)
	8,409	2,159	522	144	8,388	2,015	771	217	7617	1798
	[8,337,822]	[3,124,947]	[657,736]	[233,031]	[7,973,193]	[2,891,917]	[697,220]	[262,173]	[7,275,974]	[2,629,743]
**Hispanic**	19.2	22.5	10.7	8.2	89.3	91.9	9.2	11.4	90.8	88.6
	(18.6-19.8)	(0.21-0.24)	(9.3-12.2)	(0.07-0.10)	(87.7-90.7)	(0.74-0.83)	(7.4-11.3)	(0.10-0.13)	(88.7-92.6)	(0.87-0.90)
	8,782	3,005	781	222	9,105	2,783	955	314	8150	2469
	[11,768,281]	[6,021,375]	[1,395,216]	[490,750]	[11,797,940]	[5,530,625]	[1,119,125]	[628,315]	[10,678,814]	[4,902,310]
**Asian**	6.3	10.9	18.8	21.0	81.1	79.0	9.2	10.3	90.8	89.7
	(5.9-6.7)	(0.10-0.12)	(16.5-21.4)	(0.17-0.26)	(78.6-83.5)	(0.90-0.93)	(7.9-10.6)	(0.07-0.15)	(89.4-92.1)	(0.85-0.93)
	4,532	943	805	222	3,954	721	433	73	3,521	648
	[3,990,355]	[2,907,594]	[904,007]	[611,760]	[3,455,565]	(2,295,834)	[322,381]	[235,631]	[3,133,184]	[2,060,203]

Note: numbers in parentheses are CI=Confidence Interval, numbers in brackets are *w.n.*=weighted sample size.

[a]Chi-square among all participants; individuals who have ever been screened in their lifetime compared with women who have never been screened in their lifetime.

[b]These proportions are calculated as the percent out of the total, *% Screened*.

[c]Chi-square among individuals who have ever been screened in their lifetime; individuals who adhered to screening guidelines (recently screened) compared with individuals who have screened but not according to guidelines (not recently screened).
